# Self-reported drug utilization, health, and lifestyle factors among 70–74 year old community dwelling individuals in Western Norway. The Hordaland Health Study (HUSK)

**DOI:** 10.1186/1471-2458-6-121

**Published:** 2006-05-03

**Authors:** Mette Brekke, Steinar Hunskaar, Jørund Straand

**Affiliations:** 1 Section for General Practice, Department of General Practice and Community Health, University of Oslo, Norway; 2 Section for General Practice, Department of Public Health and Primary Health Care, University of Bergen, Norway

## Abstract

**Background:**

To examine the level and patterns of self-reported medication use (prescription and non-prescription drugs) among 70–74 year old individuals living in the community, and to explore self-reported indications for use, and factors possibly predictive of drug use.

**Methods:**

A health survey carried out in 1997–99 in the county of Hordaland (Western Norway) in the setting of a population study. A self-administered questionnaire was mailed to 4338 persons born in 1925–27, and a health check-up was offered. Drug use the previous day was reported (point prevalence). 3341 (77.0%) persons who responded, comprise the material for the analyses.

**Results:**

Between one third (males) and one quarter (females) did not take any drug the previous day. Mean number of drugs among users was 2.8 (men and women). 32% used three or more drugs and 11.5% five or more. Hypertension and other cardiovascular problems were by far the most common reasons for drug use, followed by respiratory, musculoskeletal and mental health problems. Self-reported poor health, a high Body Mass Index (BMI), and being an ex-smoker (but not currently a smoker) correlated with increasing number of drugs taken.

**Conclusion:**

Among 70–74-year old individuals living in the community no use of medication was more common than major polypharmacy (5+ drugs). Persons who had fallen ill and were put on regular medication, probably tended to quit smoking, while those who remained healthy, continued to smoke.

## Background

Elderly people are the major drug users in the community [1]. In old age, complex medical conditions are relatively frequent, which may indicate multiple drug therapy. In Sweden, drug use among elderly probands has been surveyed over many years [2]. In Norway remarkably little is known about who use drugs, and for which reasons, although whole sales statistics are available [3]. Previous Norwegian studies have been carried out on general practitioners' prescription habits [4], on drug use in nursing homes [5,6], or on the use of particular drugs, e.g. antihypertensives, psychotropics and diuretics [7].

Although appropriate drug therapy can alleviate symptoms and reduce both morbidity and mortality, the use of numerous drugs also implies increased risks of side-effects, drug-drug and drug-disease interactions, inappropriate use, and non-compliance. Physiological changes, such as decreased renal function and mental impairment, make the elderly more vulnerable to drug related harm [8]. Principally there are three major categories of sub-optimal drug use: over-use or polypharmacy, inappropriate use, and under-use [9,10]. Under-utilization may imply under-prescribing of potentially beneficial therapies because of high age [10]. Polypharmacy can be defined either as the concomitant use of multiple drugs, or the use of more medications than are clinically indicated [11]. In epidemiological studies, polypharmacy can only be assessed according to the first definition, by a cut-off value based on a simple count of medications. Although no specific number of drugs can be established to define polypharmacy, cut-off-points from three to five daily drugs per patient have been commonly used [11,9].

In the present study we explore the level and patterns of medication use (prescribed and over the counter – OTC – drugs) among 70–74 year old individuals living in the community, in relation to self-reported reasons for use, sociodemographic factors, and various health status and lifestyle parameters.

## Methods

In 1997–1999 a population based health survey was carried out in Hordaland County in western Norway (with capital city Bergen) [12]. In total, 44 342 persons were invited, among whom 4338 were born between 1925 and 1927 and were living in Bergen and three nearby communities. The survey was carried out by the National Health Screening Service, Oslo (now The Norwegian Institute of Public Health) in cooperation with the University of Bergen.

The study was approved by the Regional Committee for Medical Research Ethics and the Norwegian data Inspectorate.

A questionnaire was mailed to the invited persons, along with an invitation to a health check-up. The filled-in questionnaires were collected at the screening station, where measurements of blood pressure, height and weight, as well as blood-tests were performed. A written consent was given at the screening station.

The questionnaire included the following questions regarding drug consumption: Did you take any drug yesterday? (yes/no). If yes: report the name of the drug, the reason for taking it, and whether you usually take this drug every day (yes/no). The questionnaire provided space for reporting up to nine different drugs, and participants were asked to continue on a separate sheet if needed. It was specified that "drugs" meant all kinds of medicines: with and without prescription, herbal drugs, vitamins and minerals. Tablets, inhalators, injections, suppositories, ointments as well as droplets were included.

The drug names written by respondents were subsequently coded according to the Anatomical Therapeutic Chemical (ATC) classification system [13] and were classified to the substance (5^th^) level of the ATC codes. The reported reasons for drug use were coded according to the International Classification for Primary Care (ICPC) [14].

The questionnaire also addressed physical exercise, smoking and alcohol use, general health and depressive symptoms Table (1), as well as duration of formal education. For analysis purpose, the answers on questions on past and present smoking and alcohol use were converted to categories.

In the present paper we focus on drugs with an ATC-code. The use of non ATC-drugs will be reported elsewhere. Data from the 3341 individuals aged 70–74 who responded to the invitation (response rate 77.0%) comprise the material for this article.

Statistical analyses were performed using SPSS version 11. Bivariate comparisons were examined by chi-square test (categorical variables) and independent samples t-test (continuous variables). Bivariate relationships were computed by means of Pearson's correlation coefficient (continuous variables). Binary logistic regression analyses (enter) were carried out to examine the relationships between drug use and health status and lifestyle measures.

## Results

Out of the 3341 respondents, 948 (28.4%) reported they did not use any drugs the previous day (men: 33.1%, women: 24.6%). 2393 had taken at least one drug, in total 6 590 drugs (2719 drugs used by 985 men, 3871 drugs used by 1408 women). This corresponds to an average use of 1.97 drugs per person (men: 1.85, women: 2.07, p < 0.01) or 2.75 drugs per individual on treatment (both genders).

**Figure 1:**
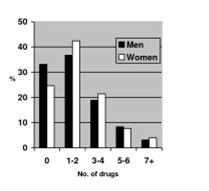
The Hordaland Health Study (HUSK), 70–74 year old individuals. Number of different ATC-drugs taken – one day prevalence

The maximum number of different drugs taken by one person on the previous day was 17. Among women, 33.0% used three or more drugs, 19.4% four or more, and 11.7% five or more drugs (Figure 1). Corresponding figures for men were 30.1, 17.6 and 11.4%, respectively. The majority reported they used the medications every day, as 84% of the 6590 drugs were taken on a daily basis.

The "top five" drug groups comprised close to one third of all medication used. These drugs were from the top: low dose aspirin (used by 15.5% of respondents), beta-blockers (15.5%), statins (13.0%), calcium channel blockers (11.7%), and ACE-antagonists (9.1%).

Reasons for use were given for 4 970 (75.4%) of the 6 590 drugs. The "top five" diagnostic reasons were: Elevated blood pressure (men: 409, women: 566, corresponding to 27.8 and 30.3% of respondents, respectively), cardiovascular problems other than hypertension [men: 514 (34.9%), women: 365, (19.5%)], respiratory tract problems, incl. allergy [men: 261(17.7%), women: 298 (16.0%)], musculo-skeletal problems [men: 68 (4.6%), women: 322 (17.2%)], and mental health problems [men: 75 (5.1%), women: 200 (10.7%)]. Table 3 shows the most frequently used drugs for the most common diagnostic categories.

### Potentially influencing factors

In bivariate analyses, female sex, low education, a poor self-reported health and feeling depressed correlated positively with increase drug use Table (4). Table 5 shows the association between lifestyle factors and drug use: Non-smokers used more drugs compared to daily cigarette smokers. However, those who had quit smoking used generally more drugs compared to all others. Abstinence from alcohol and performing no physical exercise correlated with increasing drug use, as was being overweight or obese.

An increasing use of drugs correlated positively with number of daily cigarettes smoked (Pearson's correlation coefficient: 0.058 p = 0.01), with the use of non ATC-drugs like herbal drugs, fish oil etc (Pearson's correlation coefficient: 0.074, p < 0.01), and with decreasing systolic blood pressure (Pearson's correlation coefficient: -0.041, p = 0.02), and diastolic blood pressure (Pearson's correlation coefficient: -0.090, p < 0.01) at the health check-up.

In binary logistic regression analyses, female sex (OR 1.6 {95% CI 1.04–2.5}), a self-reported poor health (OR 3.0 {1.8–5.0}), and a BMI < 27 kg/m^2 ^(OR 1.9 {1.2–3.0}) correlated positively with medication use (data not shown in tables).

## Discussion

The validity of elderly persons' self-reported drug use and reasons for use may be questioned. However, asking which drugs were taken *the previous day *(point prevalence) probably minimized recall bias [15]. Asking which drugs were actually taken also bypassed the problem of non-compliance, as we know that around 25% of prescribed drugs may not be used [16]. Data quality in our study was considered good, as drug names were generally correctly written and only few questionnaires had to be rejected because of inconsistencies. The high response rate (77%) also contributes to strengthen the validity of the results. We may assume, however, that the 23% who did not participate, would have been found to use more drugs than the respondents, as people with poor health, poor social status and unhealthy lifestyle are known to be generally over-represented among non-respondents in health surveys [17].

75% of those who had taken any drug gave an explicit reason for using it. Probably, the remaining quarter did not know or was not sure about the indications (low dose ASA, beta-blockers and estrogens were the main drug groups taken by those who gave no reason for taking them). Our figures, however, correspond well with the results in a comprehensive British interview study among people aged 65 +, where 76% knew the reason for taking their medications [16].

In our study, 28% (men: 33%, women: 25%) reported no use of ATC-drugs on the day previous to the survey. Direct comparison with other studies is complicated by differences in data collection, population sample and kind of drugs included. The previously cited British interview study, gave results almost identical with ours, as roughly one third had taken no drugs during the last 24 hours, and mean number of drugs for those on treatment was 2.8 [16]. In a Swedish study among 70-year old individuals performed in 2000, only one in five men and one in ten women reported to use no drugs at all [2]. Average number of drugs among those on treatment in Sweden was 3.3 in men and 4.0 in women, which is substantially higher than our figures. This is, however, not surprising, as the overall drug consumption in Norway (measured in daily doses per inhabitants) only reached 60% of the Swedish level in 2000 [3]. A corresponding study carried out by the Swedish authors back in 1972, showed that 40% of men and 24% of women did not use any drug. The proportion who took four or more drugs increased from 21% (1972) to 28% (2000) in men and from 30% to 41% in women [2].

A Danish study among 75-year old persons living in the community revealed that only 3% of the subjects did not take any drug, and that the average total number of drugs – prescribed and OTC – was as high as 5.4 [18]. The higher level was probably partly caused by examination of drug storages in patients' homes and thereby including drugs that were in fact not used, as total drug consumption in Denmark is slightly lower compared to Norway (close to 90%) [3]. In the Danish study, CNS drugs were the most commonly used category, compared to cardiovascular drugs in our and other studies [1,2,16,19]. 26% of the elderly Danes had taken a benzodiazepine the previous day, reflecting the relatively high use of benzodiazepines in Denmark in the late 1990s [20]. In our study, only 5% reported having taken a benzodiazepine, corresponding to the average level of use in the population in Hordaland County [3]. Other studies have shown that use of benzodiazepines increases by age [21], and we can only speculate if our results are influenced by selection bias or possible underreporting.

The lower use of drugs in our study compared to neighbouring countries probably reflects habits and preferences in the population as well as among physicians. In a population traditionally founded on fishing and agriculture like on the Western coast of Norway, it is often considered a benefit to be independent of regular medication, and to cope with occasional illnesses without drugs. Norwegian family physicians may also be more cautious than colleagues elsewhere in prescribing regular medications for elderly people, especially drugs with potential CNS side-effects. On the other hand, we may simply lag behind our neighbours in an ongoing process towards increased drug use. As the Swedish study shows, drug use among 70 year old persons increased remarkably during three decades, despite generally improved health status [2]. However, over that period, a range of new drugs have been introduced, especially for the treatment of cardiovascular risk factors [22,23]. Recently, focus has switched from concerns of unnecessary treatment to possible under-prescribing of potentially beneficial therapies to seniors [10]. Low dose aspirin, beta-blockers and lipid lowering agents were already the dominant drug groups in our sample (13% used a statin). The sales of lipid reducing agents have doubled in Norway from 1999 to 2004 [3], and recently, 13% of presumed healthy, low-risk 75-year old women in Oslo were found to use statins [24].

In our study, 31.7% had used at least three drugs, while 11.5% had taken five or more daily drugs. This is less compared to the Swedish [2] and the Danish [18] studies cited above, but equals the results of the British interview study [16]. Based on data from a Danish prescription database, Bjerrum found that on a random day, close to 5% of 70–74 year old persons used five or more prescribed drugs [11]. He also found a six-fold variation between general practices regarding the prevalence of major polypharmacy (5+ drugs), correlating – among other things – with the doctors' workload, as increasing workload implied increased prescription.

### Potentially influencing factors

Many of the factors which correlated positively with drug use in our study Tables (4 and 5) have also been identified by others: female sex, a relatively low education (especially for respiratory and cardiovascular drugs) [1] depressed mood, and a poor self-reported general health [25]. Not surprisingly, we found (in bivariate analyses) that reporting poor general health, feeling depressed, and performing no regular exercise were all strongly correlated with drug use, both with the use of any drug, with an increasing number of drugs used, as well as with the concomitant use of five or more different drugs.

It may be surprising that being a non-smoker and being abstinent from alcohol both correlated with increased use of drugs. However, having quit smoking strongly correlated to increased drug use. In the group who had smoked more than 10 cigarettes a day for 30 years or more, and who now had quit smoking, (n = 237), one out of six used five or more drugs. That former smokers were most likely to be on medication, corresponds to what has been found in other studies [25,26]. But how can we explain that heavy present smokers (more than 10 cigarettes a day for 30 years or more, n = 114) in fact used less drugs than the average? One explanation may be that people who had fallen ill and were put on regular medication, quitted smoking, while those who remained healthy in spite of their smoking habit, were more likely to continue smoking. Another probable explanation may be that a substantial proportion of heavy smokers died before reaching the age of 70–74 years [27].

A corresponding relationship may also be valid for alcohol use, but because we did not ask about quitting drinking, we could not explore this. The negative correlation between use of alcohol and medications was nevertheless weaker than for smoking. Even regular drinkers (> 10 drinking days a month or > 20 glasses of alcohol a month) used (non-significantly) less drugs compared to the rest of the sample. This corresponds with epidemiological data supporting that regular moderate alcohol consumption is associated with less morbidity and mortality [28]. An American study found no significant relationship between current alcohol use and current medication use among persons 65 and over [25]. However, in our study under-reporting of actual drinking and a selection effect implying that heavy smokers and drinkers with poor health may be overrepresented among the non-responders can not be ruled out.

## Conclusion

No use of medication was more common than major polypharmacy (5+ drugs). Among 70–74 year old individuals living in the community, between one third (males) and one fourth (females) did not use prescription or OTC drugs. Mean number of drugs among those on treatment was 2.8, and 11.5% had taken five or more drugs. Drugs for hypertension and other cardiovascular risk-factors were the most frequently used. In bivariate analyses being an ex-smoker (but not currently a smoker) correlated with drug use. Female sex (OR 1.6), being overweight (OR 1.9), and reporting a poor general health (OR 3.0), were factors that independently predicted medication use in this elderly population.

## Competing interests

The author(s) declare they have no competing interests.

## Authors' contributions

MB carried out the data analyses and drafted the article.

SH planned the study, drafted the questionnaire, and carried out the data collection.

JS planned the study, drafted the questionnaire, carried out the data collection, and participated in drafting the article.

## Pre-publication history

The pre-publication history for this paper can be accessed here:



## Figures and Tables

**Table 1:** Questions on lifestyle and health status

**Physical activity**
Which level of physical activity have you had during the last year?
• Easy exercise:*none, less than 1 hour/week, 1–2 hours/week, 3 hours or more/week*
• Hard exercise:*none, less than 1 hour/week, 1–2 hours/week, 3 hours or more/week*

**Use of alcohol**
• Are you totally abstinent from alcohol? *Yes/no*
• How many times a month do you usually drink alcohol? *Number of times:*
• How many glasses of beer, wine or spirits do you usually drink during a two-week period? *Number of glasses:*

**Smoking**
• Do you smoke cigarettes daily? *Yes/no*
• If you used to be a daily smoker, since when did you quit? *Number of years:*
• If you are or were a daily smoker, how many cigarettes do or did you usually smoke during a day? *Number of cigarettes:*
• For how many years have you been a daily smoker? *Number of years:*

**General health**
• How is your present general state of health? *Bad/not quite good/good/very good*

**Depressed mood**
• Have you felt down/depressed during the last two weeks? *Yes/a little/considerably/very much*

**Table 2:** The Hordaland Health Study (HUSK) 1997–99, 70–74 year old individuals Number of persons invited and examined^1^

Year of birth	Number invited			Number examined		Attendance^2^	
	
	*All*	*Men*	*Women*	*Men (%)*	*Women (%)*	*Bergen (%)*	*Rural areas (%)*
1925	1 401	604	797	466 (77.2)	581 (72.9)	901 (72.6)	146 (78.1)
							
1926	1 451	618	833	481 (77.8)	629 (75.5)	931 (75.3)	179 (83.3)
							
1927	1 486	646	840	526 (81.4)	658 (78.3)	1 010 (78.8)	174 (84.9)
							
Total	4 338	1 868	2 470	1 473 (78.9)	1 868 (75.6)	2 842 (76.2)	499 (82.2)

^1^All, men and women: 4 338 invited, 3 341 (77%) examined

^2 ^Both genders

**Table 3:** The Hordaland Health Study (HUSK) 1997–99, 70–74 year old individualsThe most frequently used drugs (ATC- code/therapeutic group) for the five most frequent indications. N = 3 341

Hypertension n = 975	Cardiovascular problems n = 879^1^	Respiratory problems n= 559^2^	Musculoskeletal problems n = 390^3^	Mental problems n = 275^4^
C03 Diuretics: 107	B01 AA03 Warfarin: 52	R01 Nasal preparations: 52	A12AA Calcium: 63	N05A Antipsychotics: 28
C07 Beta blocking agents: 226	B01 AC06 Low dose acetyl-salicylic acid: 313	R03 Drugs for obstructive airway diseases: 387	H02 Corticosteroids: 31	N05BA Anxiolytics: 47
C08 Calcium channel blockers: 224	C01 A Cardiac glycosides: 28	R06 Antihistamines: 54	M01A Antiinflammatory agents: 108	N05C Hypnotics: 83
C09 A+B ACE-inhibitors: 200	C01 DA Nitrates: 88		M05B Drugs affecting bone mineralization: 46	N06A Antidepressants: 94
C09 C Angiotensin II antagonists: 87	C03 Diuretics: 30			
	C07 Beta blocking agents: 147			
	C08 Calcium channel blockers: 76			
	C09 A+B ACE inhibitors: 29			
	C10 Lipid reducing agents: 36			

**Sum: **844 (87% of total)	**Sum: **799 (91% of total)	**Sum: **493 (88% of total)	**Sum: **248 (64% of total)	**Total: **252 (95% of total)

^1^angina pectoris: 159, myocardial infarction: 85, atrial fibrillation: 41 (32% of n)

^2^asthma: 358, allergy: 76 (78% of n)

^3^osteoporosis: 132, rheumatoid arthritis: 90, back pain: 25 (63% of n)

^4^sleep disturbancies: 128, depression: 57, anxiety: 56 (88% of n)

**Table 4:** The Hordaland Health Study (HUSK) 1997–99, 70–74 year old individualsDrug use related to sociodemographic factors and health status. Bivariate analyses^1,2^

***Explanatory variables***		Use of any drug % ^1^	Number of drugs used^2^	Use of five or more drugs % ^1^
	***Categories***			
Sex	*Males*	65.5	1.85	10.1
	*females*	74.4	2.07	10.7
		(p < 0.01)	(p < 0.01)	(p = 0.5)
				
Dwelling	*urban*	70.6	1.98	10.3
	*rural*	70.3	1.94	10.4
		(p = 0.9)	(p = 0.7)	(p = 0.9)
				
Education	*high*	68.3	1.92	9.6
	*low*	72.1	2.00	10.6
		(p = 0.1)	(p = 0.4)	(p = 0.5)
				
Self-reported health	*good*	62.2	1.49	4.8
	*poor*	86.3	2.93	21.4
		(p < 0.01)	(p < 0.01)	(p < 0.01)
				
Feeling depressed	*yes*	86.3	3.18	23.9
	*no*	69.2	1.88	9.3
		(p < 0.01)	(p < 0.01)	(p < 0.01)

^1^chi squared test

^2^independent samples t-test

**Table 5:** The Hordaland Health Study (HUSK) 1997–99, 70–74 year old individuals Drug use related to lifestyle factors. Bivariate analyses^1,2^

***Explanatory variables***		Use of any drug (%) ^1^	Number of drugs used^2^	Use of five or more drugs (%) ^1^
	***Categories***			
Daily cigarette smoking	*yes *(n = 482)	65.1	1.71	10.8
		71.4	2.02	9.1
	*no*	(p = 0.01)	(p < 0.01)	(p = 0.3)
Ever smoked	*yes*	70.7	2.02	11.7
		70.3	1.91	8.6
	*no *(n = 1421)	(p = 0.8)	(p = 0.1)	(p < 0.01)
Quit smoking	*yes *(n = 1347)	74.0	2.18	12.8
		68.2	1.83	8.7
	*all others*	(p < 0.01)	(p < 0.01)	(p < 0.01)
Number of cigarettes now	*1–10 *(n = 379)	66.5	1.72	9.5
	*11+ *(n = 129)	62.6	1.79	9.3
		(p = 0.2)	(p = 0.8)	(p = 0.7)
Smoked >10 cig daily for = > 30 years	*yes *(n = 357)	69.8	2.29	14.8
	*all others*	70.6	1.93	9.8
		(p = 0.8)	(p < 0.01)	(p < 0.01)
>10 cig daily for = > 30 years & smoking now	*yes(n = 114)*	60.6	1.75	8.8
	*all others*	70.9	1.98	10.4
		(p = 0.02)	(p = 0.2)	(p = 0.8)
>10 cig daily for = > 30 years & quit smoking	*yes *(n = 237)	73.5	2.56	18.1
	*all others*	70.3	1.93	9.8
		(p = 0.4)	(p < 0.01)	(p < 0.01)
Use of alcohol	*yes*	69.2	2.08	10.3
	*no *(n = 823)	73.6	1.93	10.9
		(p = 0.02)	(p = 0.1)	(p = 0.6)
Use of alcohol > 10 times/month	*yes *(n = 185)	69.4	1.96	10.8
	*no*	71.3	1.99	10.8
		(p = 0.7)	(p = 0.8)	(p = 1.0)
Use of > 10 glasses of alcohol/2 weeks	*yes *(n = 266)	67.6	1.89	10.5
	*No*	70.7	2.01	10.9
		(p = 0.3)	(p = 0.2)	(p = 0.9)
Light exercise	*yes *(n = 2 598)	69.2	1.87	9.0
	*no*	77.9	2.62	18.7
		(p < 0.01)	(p < 0.01)	(p < 0.01)
Intensive exercise	*yes *(n = 722)	64.3	1.65	6.6
	*No*	72.8	2.12	11.9
		(p < 0.01)	(p < 0.01)	(p < 0.01)
Body mass index > 27	*yes *(n = 1244)	76.7	2.25	12.8
	*No*	66.9	1.81	8.9
		(p < 0.01)	(p < 0.01)	(p < 0.01)
Body mass index > 30	*yes *(n = 462)	79.5	2.51	16.5
	*No*	69.1	1.88	9.3
		(p < 0.01)	(p < 0.01)	(p < 0.01)

^1^chi squared test

^2^independent samples t-test
